# Effects of Inorganic Salt Solutions on Vigour, Viability, Oxidative Metabolism and Germination Enzymes in Aged Cabbage and Lettuce Seeds

**DOI:** 10.3390/plants9091164

**Published:** 2020-09-09

**Authors:** Ademola Emmanuel Adetunji, Sershen, Boby Varghese, Norman W. Pammenter

**Affiliations:** 1 School of Life Sciences, University of KwaZulu-Natal, Durban 4001, South Africa; varghese@ukzn.ac.za (B.V.); pammente@ukzn.ac.za (N.W.P.); 2 Department for Biodiversity and Conservation Biology, University of the Western Cape, Private Bag X17, Bellville 7535, South Africa; sershenn@gmail.com; 3 Institute of Natural Resources, P.O. Box 100396, Scottsville 3209, South Africa

**Keywords:** controlled deterioration, lipid peroxidation, electrolysed and non-electrolysed inorganic ions, germination, vigour

## Abstract

This study assessed the potential of pre-hydration treatment with aqueous solutions (electrolysed [cathodic water; CW] and non-electrolysed) prepared from four different inorganic ion combinations: 1 mM CaCl_2_, 1 µm CaCl_2_ and 1 mM MgCl_2_ (CaMg, hereafter), 1 mM MgCl_2_ and 1 mM NaCl to invigorate controlled deteriorated (CDd) *Brassica* *oleracea* (cabbage) and *Lactuca* *sativa* (lettuce) seeds by assessing germination, vigour and biochemical markers (electrolyte leakage, lipid peroxidation products, protein carbonylation, and defence and germination associated enzymes) of oxidative stress. Additionally, the possible effects of pH of electrolysed CaMg and NaCl solutions were assessed. The inorganic salt solutions were applied to fresh seeds and seeds deteriorated to 75% viability (P75), 50% viability (P50) and 25% viability (P25); deionised water served as control. The pre-hydration treatment did not enhance normal seedling production in cabbage. However, Ca-containing and CW hydration treatments (CaCl_2_ CW, CaMg and CaMg CW [6.5], MgCl_2_ CW, NaCl CW and NaCl CW [6.5]) promoted normal seedling production of CDd lettuce seeds, while seedling vigour was enhanced by CaMg, CaMg CW (6.5), NaCl CW and NaCl CW (6.5) in CDd cabbage seeds, and CaCl_2_, CaCl_2_ CW, CaMg, CaMg CW (6.5), MgCl_2_ CW, NaCl CW and NaCl CW (6.5) in CDd lettuce seeds. The supplementation of Ca, a component of the ionised solutes, and/or the reducing potential of CW contributed to increased normal seedling production in lettuce seeds irrespective of the pH of treatment solutions or degree of deterioration. Overall, the pre-hydration treatments enhanced endogenous antioxidants leading to reduced levels of electrolyte leakage, lipid peroxidation, protein carbonylation, and enhanced germination enzyme activities in lettuce seeds. The study concluded that pre-hydration with selected inorganic salt solutions can invigorate debilitated lettuce seeds.

## 1. Introduction

At physiological maturity, characterised by low moisture content in orthodox seeds, germination and vigour are usually at the maximum level [1,2]. Mature seeds of orthodox species such as cabbage and lettuce [3,4] can be stored for long periods when dried to moisture levels as low as 5% (fresh mass [FM] basis) or less and at low temperatures in dry conditions [3]. However, the seeds do not retain the initial quality with extended storage (years), gradually deteriorating, and inevitably proceeding towards death [5,6]. As seeds deteriorate, vigour is first lost, after which comes the loss of viability [7,8]. Poor yield, which is of crucial importance in crops, is a consequence of variable combinations of low germination, delayed emergence, weak seedling and/or abnormal seedlings and poor field stand, all of which are possible when debilitated seeds are sown [6].

This age-induced physiological deterioration of seeds in storage commonly referred to as ageing is brought about by high seed moisture content, temperature and relative humidity during storage [6,9] but is also influenced by the genetic constitution of the seeds [10]. Susceptibility to ageing, and hence, the rate of deterioration in storage differs widely between species and among the varieties of related species due to factors such as storage condition and crop species diversity [11]. In several vegetable species, including cabbage [12,13] melon, pepper and lettuce [14], differences in the rates of seed deterioration during storage have been reported in relation to their storage conditions.

Ageing is intimately linked to the generation and accumulation of reactive oxygen species (ROS) that are highly reactive, toxic and capable of causing degradative reactions leading to severely damaging effects in seeds especially over an extended storage period [15,16]. Lipids, proteins, DNA and RNA, are believed to be the major ROS targets during oxidative stress [17], and the physiological lesions that result include loss of membrane integrity (through lipid peroxidation), reduced respiration, enzyme inactivation and degradation, and genetic degradation [5,9,18]. Lipid peroxidation, in particular, has been implicated in the loss of viability during storage of a number of crop seeds species [19,20] and has been shown to lead to swelling of mitochondria, increased membrane viscosity and heightened bilayer permeability (measured as increased solute leakage) [5,21]. Products of lipid oxidation can also cause DNA damage and interrupt the normal functioning of a number of cellular systems [21].

Since seed deterioration is associated with loss of structural and metabolic integrity and biochemical abnormalities, evaluating the changes in the levels of oxidative stress biomarkers such as electrolyte leakage [21], lipid peroxidation [22], protein carbonylation [23], RNA and DNA integrity [9], antioxidant capacity [20] and activities of enzymes associated with germination [8] are useful ways of identifying the factors that enhance and alleviate ageing in seeds. Attempts to measure various physiological and biochemical indicators of oxidative stress have been employed in studies focused on assessing ageing-related metabolic changes in cabbage and lettuce. In cabbage, for instance, seed ageing has been related to changes in the levels of electrolyte leakage [24]. In lettuce, seed ageing has been attributed to changes in the levels of lipid hydroperoxides [25] and volatile products such as aldehydes and alcohols [26].

Given the global need to ensure food security for a rapidly growing world population in a changing climate through ex situ seed banking, limiting seed deterioration in storage and/or reinvigoration of seeds that have deteriorated to some extent in storage has been an increasingly important research focus. Controlled pre-sowing seed hydration to a point close to, but before radicle protrusion which allows for pre-germination metabolism without actual germination [6], is one of the techniques used for enhancing seed performance. Classical seed priming protocols originally developed decades ago, include but are not limited to biopriming, hydropriming and the use of non-permeating organic osmotica (osmoconditioning). These techniques have beneficial responses such as enhanced germination rate [27], germination capacity [28], and improved seedling vigour and stress tolerance [29]. Other beneficial effects like improved membrane integrity, antiperoxidative effects, mending of cellular lesions and metabolic elimination of harmful substances induced by oxidants have also been reported [30]. In this regard, seed pre-hydration treatments using synthetic and natural compounds have been reported to be quite effective in alleviating and repairing stress-induced cellular damage in several agriculturally important species, including cabbage [31,32] and lettuce [33,34]. Additionally, seed pre-germination treatments with inorganic salt solutions like CaCl_2_ [31], MgSO_4_ [34], NaCl [35] and MgCl_2_ [36] have been shown to have restorative effects in debilitated seeds.

The exact mechanisms through which these inorganic salts protect cells against oxidative stress and in turn have restorative effects in aged seeds of various orthodox species including cabbage and lettuce when hydrated with inorganic salt solutions are not well characterised. However, Ashraf and Rauf [37] suggested that seeds take up ions from saline solutions in which they are treated leading to increased accumulation of ions in varying proportions in the different parts of seeds during germination. Uptake of ions and existing ionic competition within cells is also affected by pH level [32,38]. The competition among protons, cations and anions is of key importance for plant mineral nutrition [38] as several findings have indicated that low pH levels are associated with cation uptake inhibition, while on the other hand, there may be slight or no influence on the uptake of anion [32]. This was part of the reasoning employed by Berjak et al. [39] in their development of an invigoration approach referred to as cathodic protection which involves treating zygotic embryos [40] and seeds [41] with the cathodic fraction of an electrolysed solution of calcium (Ca) and magnesium (Mg) chloride. The reduced cathodic fraction of an electrolysed dilute ionic solution, henceforth referred to as cathodic water (CW), has a high pH and has been reported to possess strong reducing antioxidative power [39,40,42]. It has been shown to enhance germination in stored seeds of *Cucurbita maxima*, *Lycopersicon esculentum* and *Pisum sativum* seeds [41]. However, unlike other restorative seed pre-treatments that involve inorganic ions, cathodic protection has not gained popularity in germplasm banks since the mechanisms via which these solutions improve germination and vigour in stored seeds are not well characterised. Importantly, reports of studies which evaluated the restorative effects of inorganic ions suggest that their effects also appear to be species-specific [41,43].

This motivated the present study which investigated the effects of the application of a range of inorganic salt solutions (electrolysed and non-electrolysed) on germination and seedling vigour in controlled deteriorated cabbage and lettuce seeds. While previous studies have only looked at CW generated from a combination of CaCl_2_ and MgCl_2_ [39,44] and NaCl [40,42], the present study looked at the cathodic fractions of CaCl_2_ and MgCl_2_ solutions also. Where the treatment alleviated the effects of ageing on vigour and viability, established physical and biochemical markers of oxidative stress and germinability was assayed to identify the mechanism(s) through which these inorganic salts protect cells against oxidative stress. The study also investigated whether pH levels of selected electrolysed inorganic salt solutions (viz. CaMg and NaCl) influenced the effects of these inorganic salt solutions on selected physical and biochemical markers of oxidative stress and germinability.

## 2. Results

### 2.1. Effect of the Application of Inorganic Salt Solutions on % Seedling Production and Vigour of Cabbage and Lettuce Seeds

Abnormal seedling (AS) production, one of the known symptoms of ageing-induced damage in germinating seeds, were observed in the present study. The occurrence of AS ranging between 1.5% and 23.5% in cabbage and 0.5% and 19.0% in lettuce was observed only in the controlled deteriorated seeds across all inorganic salt hydration treatments (Table 1). The proportion of AS produced was reduced significantly in P25 cabbage seeds treated with CaCl_2_ CW, CaMg, CaMg CW, CaMg CW (6.5), MgCl_2_, NaCl, NaCl CW, NaCl CW (6.5), and in P50 cabbage seeds treated with NaCl CW (6.5) relative to DW-treated seeds. However, the proportion of AS produced was increased significantly in P25 lettuce seeds treated with NaCl relative to DW-treated seeds.

Percentage normal seedling production in cabbage seeds was not enhanced significantly by the application of inorganic salt solutions; rather, this was reduced in CaCl_2_- and CaCl_2_ CW-treated P50 cabbage seeds relative to seeds soaked in deionised water (DW) (Table 2). In lettuce, however, normal seedling production was increased significantly in P50 seeds treated with CaCl_2_ CW, CaMg and CaMg CW (6.5), and in P25 seeds treated with CaCl_2_ CW, CaMg, MgCl_2_ CW, NaCl CW and NaCl CW (6.5) relative to seeds soaked in DW (Table 3).

In both fresh and controlled deteriorated (P75) cabbage seeds, seedling vigour index (SVI) was not influenced significantly by soaking in inorganic salt solutions (Table S1). Seedling vigour index in P50 cabbage seeds, however, was significantly increased relative to P50 DW-treated seeds when soaked in CaMg CW (6.5) and NaCl CW (6.5). In P25 CDd cabbage seeds, SVI increased significantly relative to P25 DW-treated seeds when soaked in CaMg, NaCl CW and NaCl CW (6.5) (Table 4).

As in cabbage, in both fresh and P75 lettuce seeds SVI was not influenced significantly by inorganic salt solution application, compared with DW-treated seeds (Table S1). However, SVI in P50 lettuce seeds increased significantly relative to P50-DW-treated seeds when soaked in CaCl_2_, CaCl_2_ CW, CaMg, CaMg CW (6.5), MgCl_2_ CW, NaCl CW and NaCl CW (6.5) while SVI in P25 lettuce seeds increased significantly when treated with CaCl_2_ CW, NaCl CW and NaCl CW (6.5) compared with P25-DW-treated seeds (Table 4).

### 2.2. Effect of the Application of Inorganic Salt Solutions on Biomarkers of Oxidative Stress in Lettuce Seeds

The oxidative stress biomarkers were measured in control (fresh) and controlled deteriorated (CDd) lettuce seeds without soaking (unsoaked), and after soaking in DW; and in all those controlled deterioration (CD) × inorganic salt treatment combinations that enhanced normal seedling production (%) significantly relative to DW-treated lettuce seeds at a specific level of CD. Since the treatments did not promote % normal seedling production in cabbage seeds, these assays were not performed for this species.

**Figure 1:**
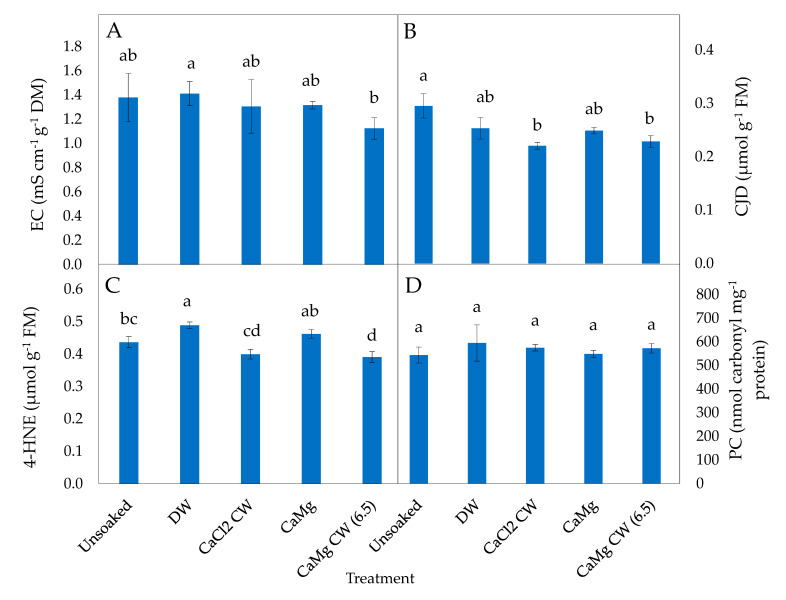
Effect of inorganic salt solution application on biomarkers of oxidative stress: (**A**) electrical conductivity (EC), (**B**) conjugated dienes (CJD), (**C**) 4-hydroxy-2-nonenal (4-HNE) and (**D**) protein carbonylation (PC) adduct, in controlled deteriorated P50 lettuce seeds subjected to no soaking or soaking in deionised water (DW), CaCl_2_ generated cathodic water (CaCl_2_ CW), CaMg, or CaMg generated cathodic water adjusted to pH 6.5 (CaMg CW [6.5]). Values represent mean ± SD (*n* = 5 for EC and *n* = 3 for all other parameters). Bars labelled with different letters indicate significant differences at *p*  <  0.05 (ANOVA).

**Figure 2:**
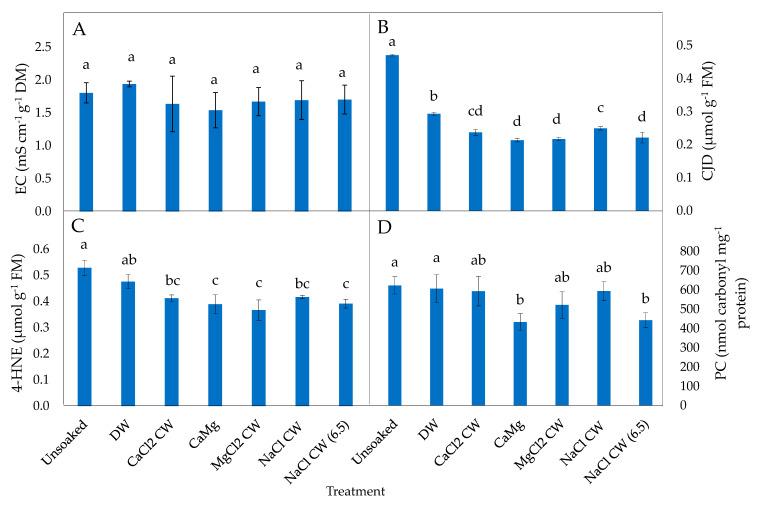
Effect of inorganic salt solution application on biomarkers of oxidative stress: (**A**) electrical conductivity (EC), (**B**) conjugated dienes (CJD), (**C**) 4-hydroxy-2-nonenal (4-HNE) and (**D**) protein carbonylation (PC) adduct, in P25 controlled deteriorated lettuce seeds subjected to no soaking or soaking in deionised water (DW), CaCl_2_ generated cathodic water (CaCl_2_ CW), CaMg, MgCl_2_ generated cathodic water (MgCl_2_ CW), NaCl generated cathodic water (NaCl CW), or NaCl generated cathodic water adjusted to pH 6.5 (NaCl CW [6.5]). Values represent mean ± SD (*n* = 5 for EC and *n* = 3 for all other parameters). Bars labelled with different letters indicate significant differences at *p*  <  0.05 (ANOVA).

Leakage levels were significantly reduced in P50 lettuce seeds treated with CaMg CW (6.5) relative to DW-treated seeds (Figure 1A), but no hydration treatments (DW and inorganic salt) led to a significant reduction in leakage in P25 lettuce seeds relative to unsoaked seeds (Figure 2A).

Conjugated dienes (CJD) levels generally reduced after all hydration treatments but this was only significant in P50 seeds soaked in CaCl_2_ CW and CaMg CW (6.5), relative to unsoaked and DW-treated P50 seeds (Figure 1B). In P25 seeds, DW and all inorganic salt soaking treatments significantly reduced CJD levels relative to unsoaked P25 seeds; the inorganic salt treatments resulted in a greater reduction in CJD levels than DM (Figure 2B).

The levels of 4-HNE were significantly increased in DW-treated P50 seeds but significantly reduced when treated with CaMg CW (6.5), relative to unsoaked P50 seeds (Figure 1C). In P25 seeds, significantly reduced levels of 4-HNE relative to unsoaked P25 seeds were estimated for all inorganic salt treatments (Figure 2C).

The inorganic salt treatments did not lead to a significant reduction in protein carbonylation (PC) levels of P50 seeds (Figure 1D), while CaMg and NaCl CW (6.5) significantly reduced PC levels in P25 seeds relative to unsoaked and DW-treated seeds (Figure 2D).

### 2.3. Effect of the Application of Inorganic Salt Solutions on Enzymatic Antioxidant Activities of Lettuce Seeds

**Figure 3:**
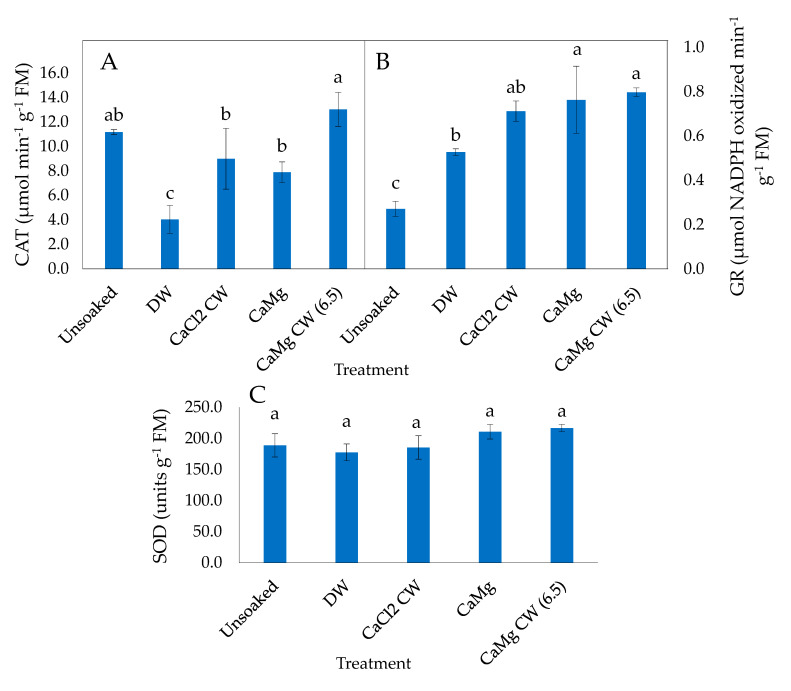
Effect of inorganic salt solution application on antioxidant enzymes activities: (**A**) catalase (CAT), (**B**) glutathione reductase (GR) and (**C**) superoxide dismutase (SOD), in P50 controlled deteriorated lettuce seeds subjected to no soaking or soaked in deionised water (DW), CaCl_2_ generated cathodic water (CaCl_2_ CW), CaMg, or CaMg generated cathodic water adjusted to pH 6.5 (CaMg CW [6.5]). Values represent mean ± SD (*n* = 3). Bars labelled with different letters indicate significant differences at *p*  <  0.05 (ANOVA).

**Figure 4:**
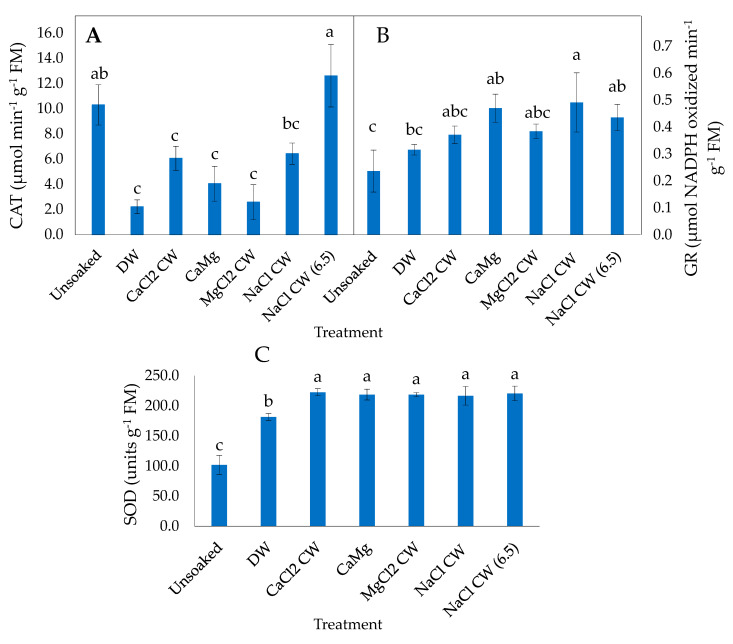
Effect of inorganic salt solution application on antioxidant enzymes activities: (**A**) catalase (CAT), (**B**) glutathione reductase (GR) and (**C**) superoxide dismutase (SOD), in P25 controlled deteriorated lettuce seeds subjected to no soaking or soaked in deionised water (DW), CaCl_2_ generated cathodic water (CaCl_2_ CW), CaMg, MgCl_2_ generated cathodic water (MgCl_2_ CW), NaCl generated cathodic water (NaCl CW), or NaCl generated cathodic water adjusted to pH 6.5 (NaCl CW [6.5]). Values represent mean ± SD (*n* = 3). Bars labelled with different letters indicate significant differences at *p*  <  0.05 (ANOVA).

In CDd P50 lettuce seeds, catalase (CAT) activity was not influenced significantly by the inorganic salt treatments relative to unsoaked seed but decreased significantly in seeds soaked in DW (Figure 3A). In P25 seeds, CAT activity was not influenced by NaCl CW and NaCl CW (6.5) soaking of seeds after deterioration, relative to the unsoaked seeds but the enzyme activity decreased significantly in seeds soaked in DW, CaCl_2_ CW, CaMg and MgCl_2_ CW (Figure 4A).

Glutathione reductase (GR) activity was increased significantly in P50 seeds in all the hydration treatments relative to the unsoaked seeds (Figure 3B). CaMg and CaMg CW (6.5) resulted in the highest GR activity among the hydration treatments. In CDd P25 seeds as well, GR activity was increased significantly by all hydration treatments (CaMg, NaCl CW and NaCl CW [6.5]), relative to unsoaked seeds (Figure 4B).

Superoxide dismutase (SOD) activity was not influenced significantly by any hydration treatment in P50 seeds relative to unsoaked seeds (Figure 3C). However, SOD activity in P25 seeds was increased significantly by all hydration treatments relative to unsoaked seeds; the inorganic salt treatments enhanced enzyme activity more than DW (Figure 4C).

### 2.4. Effect of the Application of Inorganic Salt Solutions on Germination-Related Enzymes in Lettuce Seeds

**Figure 5:**
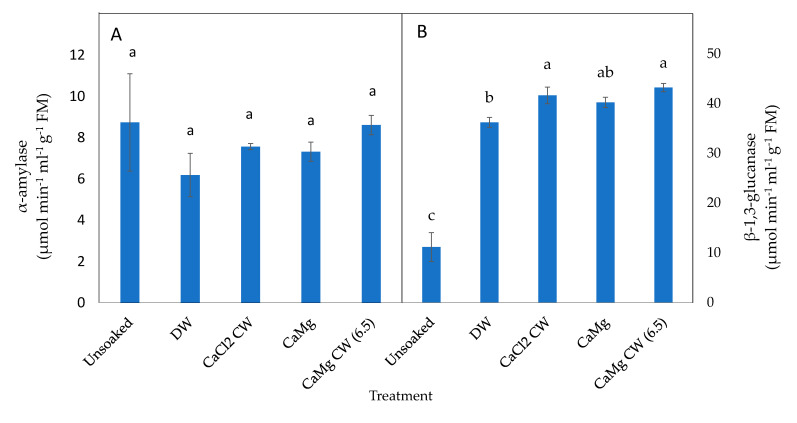
Effect of inorganic salt solution application on germination enzymes activities: (**A**) α-amylase and (**B**) β-1,3-glucanase, in P50 controlled deteriorated lettuce seeds subjected to no soaking or soaked in deionised water (DW), CaCl_2_ generated cathodic water (CaCl_2_ CW), CaMg, or CaMg generated cathodic water adjusted to pH 6.5 (CaMg CW [6.5]). Values represent mean ± SD (*n* = 3). Bars labelled with different letters indicate significant differences at *p*  <  0.05 (ANOVA).

**Figure 6:**
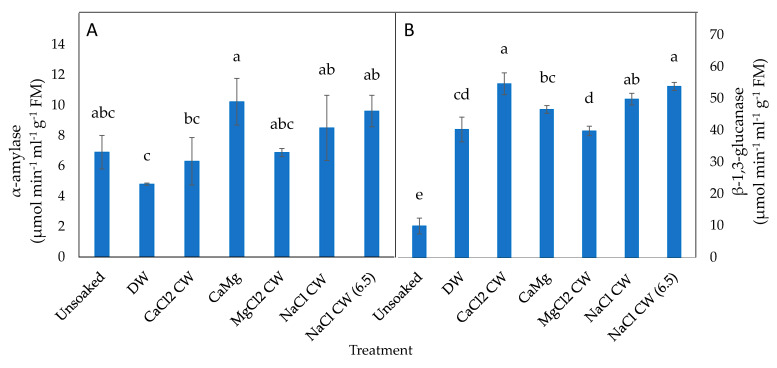
Effect of inorganic salt solution application on germination enzymes activities: (**A**) α-amylase and (**B**) β-1,3-glucanase, in P25 controlled deteriorated lettuce seeds subjected to no soaking or soaked in deionised water (DW), CaCl_2_ generated cathodic water (CaCl_2_ CW), CaMg, MgCl_2_ generated cathodic water (MgCl_2_ CW), NaCl generated cathodic water (NaCl CW), or NaCl generated cathodic water adjusted to pH 6.5 (NaCl CW [6.5]). Values represent mean ± SD (*n* = 3). Bars labelled with different letters indicate significant differences at *p*  <  0.05 (ANOVA).

The inorganic salt solutions had a promotive effect on the activities of germination enzymes in the controlled deteriorated seeds. Though the hydration treatments did not significantly enhance α-amylase activity in both P50 (Figure 5A) and P25 (Figure 6A) seeds relative to their unsoaked seeds, all hydration treatments significantly increased β-1,3-glucanase activity in P50 seeds relative to unsoaked seeds; CaCl_2_ CW and CaMg CW (6.5) soaked seeds exhibited the highest β-1,3-glucanase activity among the hydration treatments (Figure 5B).

In P25 seeds, β-1,3-glucanase activity was increased significantly by the hydration treatments relative to unsoaked seeds; CaCl_2_ CW, NaCl_2_ CW and NaCl_2_ CW (6.5) resulted in a greater rise in the enzyme activity than DW (Figure 6B).

## 3. Discussion

Studies on the invigoration of debilitated orthodox seeds have employed various seed preconditioning treatments including organic [45] and inorganic [6] hydration agents where preconditioning treatments allow deteriorated seeds to partially regain their vigour, while consequently reducing the proportion of produced AS. In the present study, the significant reduction in the proportion of AS produced in cabbage seeds treated with inorganic salt (electrolysed and non-electrolysed) solutions relative to DW-treated CDd seeds (Table 1) was mainly accompanied by a significant increase in mortality (Table S2) and no significant increase in normal seedling production relative to DW-treated seeds (Table 2). Abnormal seedling production and delayed germination resulting from loss of vigour are expressions of advanced seed deterioration. Carrozzi et al. [34] reported that abnormal seedlings production was not changed compared with control in 1 year aged lettuce (*Lactuca sativa*) seeds hydrated with an inorganic salt (MgSO_4_) solution. Tarquis and Bradford [46] also reported increased abnormal seedling production in *L*. *sativa* seeds pre-hydrated beyond 1 h after CD. In the present study, the treatment of controlled deteriorated lettuce seeds with the solution of inorganic ions (CaCl_2_ CW, CaMg, CaMg CW [6.5], MgCl_2_ CW, NaCl CW and NaCl CW [6.5]) improved normal seedling production significantly (Table 3). Comparison with the results obtained for aged cabbage seeds with the same treatments suggests a species-specific effect of these inorganic ions on post CD invigoration. These species-specific effects of specific inorganic salt solutions may be based on differences in the nature of the main oxidants and oxidant targets at the cellular level. According to Hawkins et al. [47], the nature of the predominant reactive oxidant has a substantial role to play in determining the level of impairment inflicted on their targets. Additionally, the potency of seed preconditioning agents on germination and seedling growth has been said to vary with species and the type of stress imposed [48].

However, it is worth noting that in aged lettuce seeds there were also differences in the effects of inorganic ion treatments between CD levels: CaMg CW (6.5) treatment improved normal seedling production at P50 and not in P25; MgCl_2_ CW, NaCl CW and NaCl CW (6.5) were significantly effective at P25 only (Table 3). On the other hand, CaCl_2_ CW and CaMg hydration improved normal seedling production relative to DW soaking at both P50 and P25 in lettuce. In a previous study, Gondwe et al. [41] reported that preconditioning of seeds with electrolysed and non-electrolysed solutions of CaMg improved germination in seeds of *Lycopersicon esculentum*, *Pisum sativum* and *Cucurbita maxima* after storage for 4 months at 5 °C. They attributed the beneficial effects to the strong reductive property of the electrolysed salt solution on ROS. In the present study, the enhancement of normal seedling production of lettuce seeds by the Ca containing solutions might be related to the supplementation of this divalent cation, a key second messenger that has been implicated in several oxidative stress alleviation responses [49], and/or the antioxidative potentials of CW [40,41] given that all solutions that showed a significant effect were CW solutions except where Ca was a component of the non-electrolysed inorganic salt solution. Whilst the ameliorative antioxidant effects of CW (generated using 2 mM NaCl solution) have been suggested to be related to the increased ionic product of the solvent water [42], the present study also underscores the relevance of the nature of the ionised solutes used to prepare the CW. Moreover, the mechanisms of action of the hydration treatments, perhaps, involves the fixing of ageing-induced oxidative stress alterations of ion channel activity [50] allowing for improved cellular functioning. The ion channel activity mentioned in the homeostatic modulation of the cellular ion channel is necessary to regulate cellular ion metabolic equilibrium that impacts on plant responses (adaptation to and dealing with) to various stress (abiotic and biotic) factors at the cellular level [50]. Extensive oxidative damage to receptors, transport proteins and ion channels can cause impaired cellular function [51,52]. Therefore, reactive oxidants can trigger Ca^2+^- and K^+^-permeable channels in cell membranes leading to the rise in cytosolic Ca^2+^ [53] and leakage of K^+^ [54] from the cell, respectively. As a second messenger known to be involved in several signalling responses [49,50,55], the productivity and stress tolerance (abiotic and biotic) capacity of plants are influenced by their Ca^2+^ status [48] while oxidative stress-induced leakage of K^+^ can stimulate programmed cell death [56].

Unlike the normal seedling production, which was only improved in controlled deteriorated lettuce seeds, the inorganic salt hydration treatment had a positive effect on SVI in both cabbage and lettuce seeds at P50 and P25 CD levels (Table 4). Post CD treatment with NaCl CW (6.5) showed a significant beneficial effect on SVI in both cabbage and lettuce seeds at both P50 and P25. Hydration with CaMg CW (6.5) also significantly improved the SVI in both species after CD to P50, whereas five other inorganic salt solutions (CaCl_2_, CaCl_2_ CW, CaMg, MgCl_2_ CW and NaCl CW) were beneficial in improving SVI relative to the DW treated lettuce seeds only. At P25, NaCl CW was beneficial in both species while CaMg and CaCl_2_ CW were beneficial in exhibiting higher SVI in cabbage and lettuce seeds, respectively. Treatment of aged seeds with MgSO_4_ solution has been reported to improve vigour in *L. sativa* [34] but at the time of this study, there were no previous reports of any of the inorganic ion solutions applied here shown to improve vigour in this species. Again, we attribute the beneficial effects recorded here to the ionic effect of the divalent cations. Gondwe et al. [41] reported that seed preconditioning with electrolysed and non-electrolysed solutions of CaMg improved SVI in seeds of *Lycopersicon esculentum*, *Pisum sativum* and *Cucurbita maxima*. Similarly, Iqbal and Ashraf [57] reported that seed preconditioning with CaCl_2_ solution promoted seedling vigour of *Triticum aestivum* under non-saline and even saline conditions. Other studies have shown the promotive effect of seed hydration on seedling vigour using low water potential osmotica solutions including NaCl (1 mM) in *Capsicum annuum* seeds subjected to salt stress [35], CaCl_2_ in *Brassica napus* seeds subjected to accelerated ageing [31] and MgCl_2_ [36] in *Brassica oleracea* seeds under standard germination conditions. However, almost all these studies have not gone to the extent of trying to understand the underlying biochemical changes responsible for improved normal seedling production and SVI due to inorganic salt hydration treatments.

On this note, the present study demonstrated that treatment with beneficial inorganic salt solutions significantly influenced some biochemical markers of oxidative stress in controlled deteriorated lettuce seeds. In effect, CaMg CW (6.5) significantly reduced EC levels in P50 lettuce seeds (Figure 1A) indicating that the inorganic salt hydration treatment had a restorative effect on lettuce seed membranes and reduced the heightened leakage of ions traditionally associated with ageing in seeds. Inorganic ions have been suggested to be involved in protecting cytoskeleton or cell membrane from injury [58] due to their promotive effect on the structure and stability of membrane lipid bilayer [58]. In addition, cation-enhanced interactions between lipids allow for closeness between lipid molecules thereby increasing membrane density [58]. In previous studies, Abdolahi et al. [31] reported that KH_2_PO_4_ solution lessened EC in three cultivars (RGS, ‘Hyola 401′ and ‘Pacific’) of *Brassica napus* seeds subjected to accelerated ageing for different durations (48 and 96 h). However, they also showed that CaCl_2_ solution performed otherwise in the same study, while Sathish and Sundareswaran [59] found CaCl_2_, KH_2_PO_4_ and KNO_3_ inorganic salt hydration treatments to have no significant effect on the EC of three genotypes (UMI 61, UMI 285 and COH[M] 5) of aged *Zea mays* seeds. Overall, it appears that seed hydration treatments may have a promotive effect or be ineffective or even detrimental in some species [46].

The basis of exposing aged seeds to different soaking treatments is generally to enhance recovery from and/or reduce ageing-induced peroxidative changes. Lipid peroxidation products (CJD and 4-HNE) levels were reduced significantly by CaMg CW (6.5) in P50 lettuce seeds, and CaCl_2_ CW reduced CJD levels in both P50 (Figure 1B) and P25 (Figure 2B) in this species. In P25 seeds of the same species, five inorganic salt treatment solutions (viz. CaCl_2_ CW, CaMg, MgCl_2_ CW, NaCl CW and NaCl CW [6.5]) significantly reduced CJD and 4-HNE while DW was only effective in CJD reduction (Figure 3B,C). Previous studies have reported that seed preconditioning led to the reduction of lipid peroxidation products in different plant organs and under various stress conditions. Chowdhury and Choudhuri [60], for instance, reported CaCl_2_ seed pre-hydration to reduce lipid peroxidation (malondialdehyde [MDA]) in water-stressed seeds of *Corchorus capsularis* and *C. olitorius*. Additionally, Khorshidi and Nojavan [61] reported reduced levels of MDA in the roots and shoots of cold stressed *Zea mays* seedlings produced from seeds pre-treated with CaCl*_2_* solution. Those authors suggested that the inorganic salt hydration treatment enhanced antioxidative enzymes activity, thereby leading to reduced cold stress injury. Seed pre-soaking in water also reduced total peroxide and MDA levels in *Momordica charantia* seeds [62]. The present study shows that in addition to the strong reductive properties of CW, the inorganic ions in the CW might be improving hydrolytic and antiperoxidative enzyme activities to offset lipid peroxidation effects [63]. Seed quality enhancement by seed hydration treatments has been ascribed to lessened ROS-mediated lipid peroxidation [64].

As products of stress-induced lipid peroxidation, certain reactive carbonyl species (*α*,*β*-unsaturated ketones and aldehydes) have been implicated in the mediation of ROS signals leading to the modification of proteins [65]. The resultant oxidation of proteins leads to alterations in protein structural and functional properties [66]. Specific germination enzymes can also be carbonylated [23,67], resulting in loss of seed vigour. In the present study, the product of oxidative modification of proteins, measured as PC, was lowered significantly by CaMg and NaCl CW (6.5) in P25 lettuce seeds only (Figure 2D). This effect may be ascribed to the direct scavenging of ROS by CW and/or enhanced activities of endogenous antioxidative enzymes. Though not assessed in the present study, perhaps, the action of the inorganic salt hydration treatment might involve enhancement of injurious oxidant and carbonyl scavengers that constitute the non-enzymatic antioxidant defence system. As reported by Dell’Aquila [68], ageing stress led to the oxidative degradation of proteins in *Triticum durum* seeds. Oxidised (carbonylated) proteins are targeted for proteolysis [69], and if not degraded, can constitute a large molecular weight assemblage, which accrues with age [23]. The intracellular proteolysis resulting from the oxidation of specific amino acids may be involved in the stress-induced restructuring of plant metabolic process, as some reports have indicated that some species are more prone to proteolytic reactions than the others when exposed to stress [69].

High antioxidant activity is known to defend plants from oxidative damage accumulated due to oxidative stress thereby increasing their survival under stress conditions [70,71].This is achieved through the activities of free radicals and ROS detoxifying antioxidative enzymes such as CAT, GR, SOD, amongst others, which scavenge oxidants capable of attacking amino acids, proteins and lipids that are essential for cell functioning and integrity [70]. Reduced activities of antioxidant enzymes such as CAT, GR, SOD and POX in aged seeds cause lowered seed respiratory competency, and energy supply resulting in loss of viability [72]. The results of the present study show that the post CD hydration treatments did not have any significant effect on CAT activities but enhanced GR and SOD activity in lettuce seeds (Figure 3 A–C and Figure 4A–C). More specifically, post CD soaking of seeds with DW and all tested inorganic salt solutions enhanced GR and SOD activity in P50 and P25 lettuce seeds, respectively, while CaMg, NaCl CW and NaCl CW (6.5) improved GR activity in P25 lettuce seeds only. The direct antioxidative properties of CW on ROS and the stimulation of endogenous antioxidant enzymes activities by the inorganic salt hydration treatments may, therefore, have contributed to the improvement in normal seedling production and seedling vigour observed in lettuce. Khorshidi and Nojavan [61] stated that cations, particularly Ca^2+^, can enhance the activities of most enzymatic antioxidants. At the same time, it is worth mentioning that soaking in water can also result in a rise in antioxidant enzymes, including GR and SOD activities (*Momordica charantia* [62]).

Seed germination enzymes are critical in the early growth stages of a germinating seed; most importantly, some of them are responsible for solubilising excess food stored as protein, lipid and starch to release energy for embryo development [43,73]. During germination, the process of mobilisation of stored food to the embryo is ubiquitous [43] but can be disturbed when already damaged seeds are exposed to stress conditions during germination, thereby exacerbating the damage. The degree of such disturbance depends on the levels and efficacy of germination associated enzymes involved in chemical reserve hydrolysis. It is envisaged that hydration of CDd seeds with inorganic salt solutions would have enhanced the germination enzymes activity, thereby contributing to organic substances mobilisation to various embryonic regions resulting in better germination and normal seedling establishment [43]. Hence, a rise in germination enzyme activity can result in improved vigour and viability. The present study revealed that the inorganic salt hydration treatments (electrolysed or non-electrolysed) did not influence α-amylase activity (Figure 5A and Figure 6A) significantly; however, all inorganic salt solutions examined as well as DW enhanced β-1,3-glucanase activity in both P50 and P25 lettuce seeds (Figure 5B and Figure 6B). β-glucanases function in the hydrolysis of β-linked glucans and their activities can be regulated by abiotic and biotic stresses [74]. As various forms of β-glucans contribute to cell wall composition, the involvement of β-glucanases in processes that might alter cell walls structure and function is reasonable. There are suggestions that they influence cell wall matrix composition and viscoelastic properties which contribute to cell expansion [74,75]. The results of the present study, therefore, suggest that enhancement of β-1,3-ucanase activity by the hydration treatments may have contributed to the increased production of normal seedlings and vigour of debilitated lettuce seeds.

The results of the present study indicated that CaMg CW (6.5) and NaCl CW (6.5) were beneficial in terms of promoting normal seedling production in CDd lettuce seeds, seedling vigor in CDd cabbage and lettuce seeds, ameliorating oxidative damage and enhancing antioxidative and germination-related enzyme activities in CDd lettuce seeds. There are reports of vegetable species (e.g., lettuce) growing better in solution-culture when pH is in the range of 7 [76]; however, the lack of information on the influence of pH on the effects of antioxidant-based soaking solutions on aged seeds suggests that this may represent a potential future area of research.

## 4. Materials and Methods

### 4.1. Seed Material

*Brassica oleracea* L. (cabbage, ‘Glory of Enkhuizen’) and *Lactuca sativa* L. (lettuce, ‘Great Lakes’) seeds supplied in airtight plastic bags were bought from McDonalds Seeds (Pietermaritzburg, South Africa), stored at 4 °C and used in less than 3 months of storage after purchase.

### 4.2. Seed Vigour Assessment

An initial germination and vigour test (three trials of *n* = 25) was conducted for every seed lot before being exposed to the CD and application of inorganic salt solutions as described below. Only high vigour seed lots (germination >85% in cabbage and germination >95% in lettuce 2 days after sowing [DAS]) were used for assays that follow.

### 4.3. Controlled Deterioration

**Figure 7:**
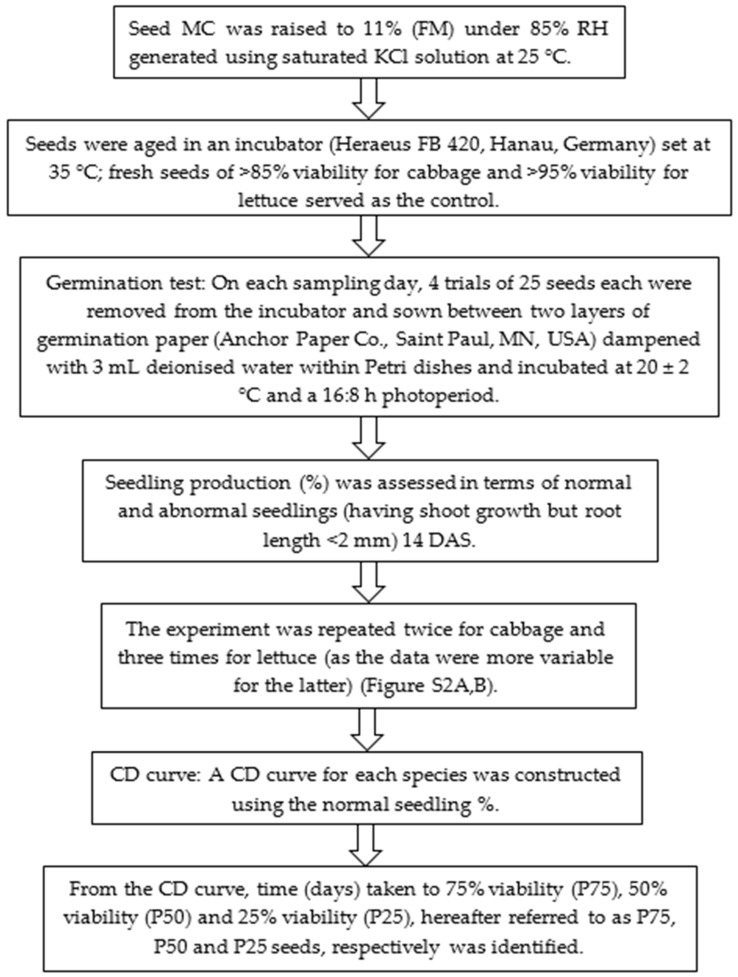
Steps followed to generate controlled deterioration (CD) curves used in this study (modified after Tekrony [78]). Days after sowing, DAS; controlled deterioration, CD.

Seed moisture content (MC, % fresh mass [FM]) was determined using the low-temperature oven method [77], while CD was performed according to Tekrony [78], with slight modifications (Figure 7) as shown below.

### 4.4. Preparation of Inorganic Salt Solutions

In this study, seeds were treated with non-electrolysed and the electrolysed CW (after Berjak et al. [39] of four inorganic salt solutions, viz. CaCl_2_, CaMg [79], MgCl_2_ and NaCl (Table 5).

To generate CW from any of the inorganic salt solutions used here, the solution was electrolysed using a BioRad™ Powerpac (BioRad, Hercules, CA, USA) equipped with two platinum electrodes. Each electrode was immersed in a 250 mL glass beaker containing 200 mL of the inorganic salt solution. Charge balance was maintained within the internal circuit by an agar-based salt bridge (30% KCl and 3% agar bacteriological), after which the solution was electrolysed at 60 V potential difference and 400 mA for 60 min. As mentioned above, only the cathodic fraction was used for seed treatments, within 24 h of preparation [80].

To evaluate the possible influence, if any, of pH given that electrolysis of ionic solutions can raise the pH of the cathodic fraction significantly (in the range of 9 and above [42]) after 60 min (the duration used in the present study), the pH of CaMg and NaCl solutions (randomly selected) was adjusted to 6.5 with 1 M HCl solution.

### 4.5. Application of Inorganic Salt Hydration Treatment

Firstly, a hydration curve was generated for each species by hydrating (using 3 mL DW) three replicates of 25 seeds (cabbage, 0.08 g; lettuce, 0.02 g) placed between two discs of germination paper (Anchor Paper Co., Saint Paul, MN, USA), one below and the other above, in Petri dishes (90 × 15 mm) at laboratory temperature of 23 ± 2 °C. The Petri dishes were covered and sealed with parafilm to prevent drying out. They were left for imbibition on a benchtop shaker (Labcon SPO 15-MP orbital, Maraisburg, South Africa) set at 100 rpm. At 2-h intervals, the seeds were blotted with a paper towel, weighed and returned to the moistened Petri dish. The process was repeated until the first signs of radicle protrusion (2 mm long). The data was then used to identify the hydration time required to reach the early germination phase (phase 2) but before radicle protrusion [33]. Once generated, these curves indicated that 8 h for cabbage and 6 h for lettuce were suitable hydration times for the seed treatments (Figure S1).

For treatment applications, four trials of *n* = 25 of fresh and controlled deteriorated (P75, P50 and P25) seeds of both species were hydrated in 3 mL of DW and seed treatment solutions (Table 5), as described for the hydration curve, for a period of 8 and 6 h for cabbage and lettuce, respectively. The seed exterior was then dried with blotting paper and seeds were germinated as outlined under CD experiments (Figure 7). The seeds used for this experiment were hydrated without drying back as there have been reports of delayed germination and/or emergence when primed seeds are dried back relative to primed but not dried back seeds owing to the extra time needed for rehydration [81]. Seedling production (normal and abnormal) and seedling vigour index were assessed 14 DAS.

#### 4.5.1. Assessment of Oxidative Stress and Germinability Biomarkers

Where inorganic salt hydration treatment of seeds enhanced normal seedling production relative to the control significantly (*p* < 0.05, ANOVA), estimation of electrical conductivity (EC) and other biomarkers of oxidative stress and germinability was performed. For comparison, unsoaked and DW-soaked seeds were used as controls. All soaked seeds were blotted before use in any assay.

##### Electrolyte Conductivity

Electrolyte conductivity was evaluated with a multi-cell (CM100-2) conductivity meter (Reid & Associates, Durban, South Africa) following the method of Sershen et al. [82] with slight modifications. Individual seeds (*n* = 5) were soaked in 2 mL of inorganic salt solution or DW for 8 h. The EC was then determined using 1.5 mL of the solution. Thereafter, the dry mass (DM) of each seed was determined. Leakage was represented as average conductivity after 8 h less the conductivity of DW and the respective inorganic salt solutions used as blanks, in mS cm^−1^ g^−1^ DM.

##### Conjugated Dienes

This was estimated following the method described by Parkhey et al. [22]. Seeds (three replicates of 0.25 g each) were homogenised in 4 mL of methanol containing 0.02% (*w*/*v*) EDTA, 1% (*w*/*v*) NaCl and 2 mL chloroform. The homogenate was centrifuged (Model J-E, Beckman Coulter Avanti^®^, La Brea, CA, USA) for 20 min at 11,000 *g* and 4 °C. From the chloroform phase, a 100 µL aliquot was taken and dried under a nitrogen gas stream. This was solubilised in 2 mL ethanol and the absorbance measured at 234 nm. Conjugated diene levels were computed with an extinction coefficient of 25 mM^−1^ cm^−1^ and expressed in µmol g^−1^ fresh mass (FM).

##### 4-Hydroxy-2-Nonenal (4-HNE)

This was estimated following the method described by Parkhey et al. [22]. Three replicates of 0.25 g seeds each were ground with 2 mL of 0.2 M borate buffer (pH 7.4) and 750 µL of 10% (*w*/*v*) TCA. The homogenate was centrifuged (Model J-E, Beckman Coulter Avanti^®^, La Brea, CA, USA) for 20 min at 11,000 *g* and 4 °C. An aliquot of 1 mL was then added to 1% (*w*/*v*) DNPH (1 mL) dissolved in 0.5 M hydrochloric acid and incubated for 2 h at room temperature. This was precipitated with hexane (2 mL) and the precipitate was dried under a nitrogen gas stream. This was solubilised in methanol (2 mL), and absorbance was measured at 350 nm. The level of 4-HNE was computed with an extinction coefficient of 13,750 M^−1^ cm^−1^ and expressed in mmol g^−1^ FM.

##### Protein Carbonylation Evaluation

Protein extraction was carried out according to Juszczuk et al. [66]. Total protein content was estimated following Bradford [83] and brought to a concentration of 10 mg mL^−1^ using DW. Protein carbonylation was estimated using the spectrophotometric method described by Augustyniak et al. [84] with slight modifications. A solution (100 µL) of 10 mM DNPH in HCl (2.5 M) was mixed with 100 µL of protein. The solution was vortexed and kept in the dark at room temperature for 10 min. Then, 30 µL of TCA solution was added, and the solution was kept on ice for 5 min and centrifuged (Model J-E, Beckman Coulter Avanti^®^, La Brea, CA, USA) for 10 min at 13,000 *g*. The supernatant was discarded, and the pellet solubilised in 500 µL of chilled acetone and sonicated for 30 s. This mixture was kept for 5 min at −20 °C and centrifuged (Model J-E, Beckman Coulter Avanti^®^, La Brea, CA, USA) for 2 min at 13,000 *g* and 4 °C. The pellet was washed with acetone and sonicated briefly in 6 M guanidine hydrochloride (200 µL), and the absorbance read at 370 nm against a guanidine hydrochloride blank. Protein carbonyl content was computed with a molar coefficient of 22,000 M^−1^ cm^−1^ and expressed in nM carbonyl mg^−1^ protein.

#### 4.5.2. Enzymatic Antioxidant Activity

Extraction of enzymes was performed following Farrant et al. [85]. Three replicates of 0.25 g seeds each were ground with liquid nitrogen (LN) and suspended in 4 mL of 0.1 M sodium phosphate buffer (pH7.8), containing 0.1 mM EDTA, 2 mM dithiothreitol, 1.25 mM PEG 4000 and 0.1 g PVP. This was then centrifuged (Model J-E, Beckman Coulter Avanti^®^, La Brea, CA, USA) at 4 °C for 30 min at 16,000× *g*. The supernatant was separated and used to estimate CAT, GR and SOD as described below.

##### Catalase Activity

Catalase activity was estimated according to Claiborne [86]. The assay mixture containing 37.5 mM of potassium phosphate buffer (pH 7.0), 10 mM of H_2_O_2_ and 100 µL of enzyme extract was prepared in the dark. A UV-Vis spectrophotometer (Shimadzu UV-2600, Kyoto, Japan) was used to measure the breakdown of H_2_O_2_ as a decline in absorbance at 240 nm. The activity was computed with an extinction coefficient of 0.0436 mM^−1^ cm^−1^ and expressed in µmol H_2_O_2_ decomposed min^−1^ g^−1^ FM.

##### Glutathione Reductase

Glutathione reductase activity was assayed using the method of Esterbauer and Grill [87] as modified by Farrant et al. [85]. The assay mixture contained 50 mM potassium phosphate buffer (pH 7.8), 3 mM MgCl_2_, 10 mM GSSG, 0.5 mM NADPH and 50 μL of enzyme extract. The absorbance was measured at 340 nm against potassium phosphate buffer blank. The activity was computed using an extinction coefficient of 6.22 mM^−1^ cm^−1^ and expressed in μmol NADPH oxidised min^−1^ g^−1^ FM.

##### Superoxide Dismutase

The estimation of superoxide dismutase (SOD) was based on Beauchamp and Fridovich [88] as modified by Varghese et al. [89]. The assay mixture comprised 50 mM sodium phosphate buffer, 1.17 µM riboflavin, 0.01 M methionine, 0.056 mM nitroblue tetrazolium (NBT) and 100 µL extract. The assay mixture was pipetted into a cuvette, which was placed on an aluminium foil plate and illuminated with 55-W white fluorescent light (Philips, Johannesburg, South Africa). Enzyme activity was estimated with the enzymatic inhibition of NBT photoreduction. A unit SOD corresponded to 50% inhibition of NBT photoreduction by the enzyme. The activity was expressed in units of SOD g^−1^ FM.

#### 4.5.3. Germination Associated Enzymes

##### α-Amylase Activity

Enzymes were extracted according to Biswas et al. [90] with slight modifications by Farashah et al. [91]. Three replicates of 0.25 g seeds were ground with LN and suspended in 2.5 mL of chilled 0.1 M phosphate buffer (pH 7.2). The homogenate was centrifuged (Model J-E, Beckman Coulter Avanti^®^, La Brea, CA, USA) for 25 min at 10,000 rpm and 4 °C and the supernatant decanted. After that, α-amylase activity was measured according to Bernfeld [92] and Baker [93] with slight modifications by Farashah et al. [91]. Starch solution (1% *w*/*v*), prepared in phosphate buffer, was added to 50 µL extract. The reaction mixture was kept at 37 °C for 30 min and mixed with 100 µL dinitrosalicylic acid reagent. The mixture was boiled for 10 min, and DW (350 µL) was added to it. The absorbance of this mixture was measured at 540 nm. The activity was defined as the amount of enzyme that liberated 1 µmol of reducing sugar (maltose) min^−1^ ml^−1^ g^−1^ FM.

##### β-1,3-Glucanase Activity

The enzyme was extracted using the method described by Farashah et al. [91]. Three replicates of 0.25 g seeds were ground with LN and suspended in 2.5 mL of 15 mM sodium acetate buffer (pH 5.5). This was centrifuged (Model J-E, Beckman Coulter Avanti^®^, La Brea, CA, USA) for 5 min at 10,000 rpm and 4 °C. The β-1,3-glucanase activity was estimated as described by Celestino et al. [94] with slight modifications. The assay mixture comprised of laminarin (1% *w*/*v*) dissolved in 100 mM sodium acetate buffer (pH 5.0) and 50 µL of extract. This was kept for 30 min at 50 °C before DNS reagent (300 µL) was added to it. The mixture was then boiled for 5 min, and the absorbance measured at 550 nm. The activity was defined as the amount of enzyme that liberated 1 µmol of reducing sugar (glucose) min^−1^ ml^−1^ g^−1^ FM.

### 4.6. Statistical Analysis

Data were analysed with IBM SPSS Statistics (Ver. 26.0. Armonk, NY, USA). Normality was tested for using a Shapiro–Wilk test. Percentage data were arcsine transformed before analysis. To test for significance, normal seedling (%) data, vigour index and biochemical parameters were subjected to ANOVA where data were parametric. Separation of means was done using a Tukey post-hoc test. Where data did not satisfy the assumptions of ANOVA, even after transformation, a Kruskal–Wallis test was used. Differences were regarded as significant at 0.05 level of probability.

## 5. Conclusions

Overall, the exogenous application of inorganic salt solutions as pre-hydration treatments for orthodox seeds invigoration was beneficial in improving normal seedling production of lettuce and seedling vigour of both cabbage and lettuce seeds subjected to CD. In this regard, CaCl_2_ CW, CaMg, CaMg CW (6.5), MgCl_2_ CW, NaCl CW and NaCl CW (6.5) are the best pre-hydration treatments having promotive effects on CDd lettuce seeds in terms of normal seedling production. In terms of seedling vigour, CaMg, CaMg CW (6.5), NaCl CW and NaCl CW (6.5) have promotive effect in CDd cabbage seeds, and CaCl_2_, CaCl_2_ CW, CaMg, CaMg CW (6.5), MgCl_2_ CW, NaCl CW and NaCl CW (6.5) in CDd lettuce seeds.

The promotive effects of the inorganic salt pre-treatment solutions appear to be based on the stimulation of antioxidative and germination-related enzymes activities in lettuce, which in turn enhances energy metabolisms, early mobilisation of stored food and endosperm weakening. At the same time, the promotive effect on cabbage seedling vigour might be purely a functional nutrient [95,96] effect for growth-related events.

The findings of the present study also suggest that the essentiality of calcium ions (Ca^2+^) in conjunction with the plausible restorative strength of CW contribute to the substantial increase in normal seedling production in lettuce, irrespective of the pH of the treatment solutions or extent of deterioration (for P50 and P25 at least).

Importantly, the results suggest that the inorganic salt hydration treatment effects are based on the direct reductive power of CW and the stimulation of endogenous antioxidants (GR and SOD), which in turn mitigate the effects of stress-induced oxidative injury (e.g., reduced EC, lipid peroxidation and protein carbonylation) in lettuce seeds. Additionally, pre-hydration treatment with specific inorganic ion solutions (and even DW) enhance germination enzyme activities in lettuce seeds.

In terms of recommendations, seed bank practitioners should note that the effects of the inorganic salt hydration treatments appear to be dependent on the nature of the main oxidants and oxidant targets in specific species. For example, the oxidant(s) and oxidant target(s) in cabbage appear to be different from that in lettuce, and by implication, the promotive effect of specific hydration treatments differed between species.

The results also suggest that certain hydration treatments may improve cellular function in deteriorated seeds by influencing intracellular Ca^2+^ and K^+^ levels, and this should form part of future studies.

## Figures and Tables

**Table 1:** Effect of the application of inorganic salt solutions on abnormal seedling production (%) in controlled deteriorated cabbage and lettuce seeds.

Hydration Treatments	AS (%) for P75	AS (%) for P50	AS (%) for P25	AS (%) for P75	AS (%) for P50	AS (%) for P25
	Cabbage Seeds	Cabbage Seeds	Cabbage Seeds	Lettuce Seeds	Lettuce Seeds	Lettuce Seeds
DW	11.00 ± 8.75 ^NS^	18.50 ± 10.89 ^a^	20.00 ± 5.24 ^a^	6.50 ± 6.74 ^NS^	11.50 ± 9.18 ^NS^	9.00 ± 4.14 ^b^
CaCl_2_	5.50 ± 5.21 ^NS^	9.00 ± 8.49 ^NS^	12.50 ± 8.67 ^NS^	7.00 ± 5.13 ^NS^	9.00 ± 7.33 ^NS^	13.00 ± 5.95 ^NS^
CaCl_2_ CW	1.50 ± 2.98 ^NS^	7.50 ± 6.95 ^NS^	7.50 ±9.18 ^b^	3.00 ± 4.66 ^NS^	4.00 ± 5.66 ^NS^	7.00 ± 7.63 ^NS^
CaMg	6.50 ± 6.39 ^NS^	12.50 ± 3.34 ^NS^	8.00 ± 5.24 ^b^	3.00 ± 04.66 ^NS^	10.50 ± 5.21 ^NS^	8.50 ± 6.21 ^NS^
CaMg CW	5.00 ± 5.95 ^NS^	17.50 ± 7.39 ^NS^	6.00 ± 5.24 ^b^	3.50 ± 4.50 ^NS^	5.50 ± 7.07 ^NS^	11.00 ± 4.66 ^NS^
CaMg CW (6.5)	7.00 ± 5.55 ^NS^	14.00 ± 4.78 ^NS^	10.00 ± 4.78 ^b^	3.00 ± 3.55 ^NS^	4.00 ± 3.02 ^NS^	8.00 ± 6.76 ^NS^
MgCl_2_	6.50 ± 6.39 ^NS^	10.50 ± 4.24 ^NS^	8.00 ± 4.78 ^b^	4.00 ± 6.05 ^NS^	2.50 ± 5.63 ^NS^	12.00 ± 5.66 ^NS^
MgCl_2_ CW	7.00 ± 6.32 ^NS^	13.50 ± 5.21 ^NS^	14.00 ± 5.66 ^NS^	1.50 ± 2.98 ^NS^	6.50 ± 6.39 ^NS^	11.00 ± 5.13 ^NS^
NaCl	16.00 ± 8.00 ^NS^	23.50 ± 6.57 ^NS^	4.75 ± 1.58 ^b^	2.75 ± 1.83 ^NS^	18.00 ± 3.70 ^NS^	19.00 ± 4.14 ^a^
NaCl CW	9.50 ± 5.63 ^NS^	7.50 ± 4.14 ^NS^	9.00 ± 8.21 ^b^	0.50 ± 1.41 ^NS^	8.50 ± 5.83 ^NS^	10.50 ± 6.02 ^NS^
NaCl CW (6.5)	8.00 ± 4.78 ^NS^	6.50 ± 5.95 ^b^	9.00 ± 5.95 ^b^	5.00 ± 4.14 ^NS^	6.00 ± 5.66 ^NS^	5.00 ± 6.32 ^NS^

Values represent mean ± SD (4 × *n* = 25) % abnormal seedling production of cabbage and lettuce seeds exposed to deionised water (DW) and inorganic salt hydration treatments after CD. Values labelled with different letters are significantly different (*p* < 0.05, ANOVA) when compared across hydration treatments within each CD level. Cathodic water, CW; cathodic water adjusted to pH 6.5, CW (6.5); controlled deterioration, CD; abnormal seedling, AS; NS: not significantly different from value obtained with DW and therefore not considered in statistical comparisons.

**Table 2:** Effect of the application of inorganic salt solutions on normal seedling production (%) in fresh and controlled deteriorated (CDd) cabbage seeds.

Hydration Treatments	% Normal Seedlings for Fresh Seeds	% Normal Seedlings for CDd (P75) Seeds	% Normal Seedlings for CDd (P50) Seeds	% Normal Seedlings for CDd (P25) Seeds
DW	89.00 ± 9.26 ^NS^	72.00 ± 14.81 ^NS^	43.00 ± 10.64 ^a^	20.00 ± 3.02 ^NS^
CaCl_2_	94.00 ± 6.76 ^NS^	71.00 ± 9.74 ^NS^	26.00 ± 7.09 ^b^	17.00 ± 7.33 ^NS^
CaCl_2_ CW	94.50 ± 5.21 ^NS^	78.50 ± 10.01 ^NS^	21.50 ± 10.24 ^b^	19.00 ± 6.32 ^NS^
CaMg	92.50 ± 7.54 ^NS^	63.50 ± 12.91 ^NS^	36.00 ± 6.05 ^NS^	23.50 ± 10.13 ^NS^
CaMg CW	91.50 ± 9.43 ^NS^	72.5 ± 10.99 ^NS^	49.50 ± 9.30 ^NS^	22.00 ± 3.70 ^NS^
CaMg CW (6.5)	96.50 ± 5.83 ^NS^	69.50 ± 12.46 ^NS^	48.00 ± 7.41 ^NS^	26.50 ± 3.66 ^NS^
MgCl_2_	95.00 ± 7.01 ^NS^	78.00 ± 8.28 ^NS^	35.00 ± 9.50 ^NS^	14.50 ± 8.26 ^NS^
MgCl_2_ CW	92.50 ± 9.90 ^NS^	75.50+11.60 ^NS^	40.00 ± 7.71 ^NS^	21.50 ± 10.68 ^NS^
NaCl	93.50 ± 5.47 ^NS^	59.00 ± 4.14 ^NS^	48.00 ± 9.80 ^NS^	24.50 ± 7.84 ^NS^
NaCl CW	95.50 ± 4.99 ^NS^	75.00 ± 5.13 ^NS^	34.00 ± 10.25 ^NS^	25.50 ± 8.26 ^NS^
NaCl CW (6.5)	94.00 ± 8.00 ^NS^	69.50 ± 11.70 ^NS^	48.50 ± 6.91 ^NS^	32.00 ± 9.32 ^NS^

Values represent mean ± SD (4 × *n* = 25) % normal seedling production of the control (fresh seeds soaked in deionised water [DW] and all the inorganic salt solutions), and CDd (P75, P50 and P25) cabbage seeds exposed to DW and inorganic salt solutions. Values labelled with different letters are significantly different (*p* < 0.05, ANOVA) when compared across hydration treatments at P50. Cathodic water, CW; cathodic water adjusted to pH 6.5, CW (6.5); NS: not significantly different from value obtained with DW and therefore not considered in statistical comparisons.

**Table 3:** Effect of the application of inorganic salt solutions on normal seedling production (%) in fresh and controlled deteriorated (CDd) lettuce seeds.

Hydration Treatments	% Normal Seedling for Fresh Seeds	% Normal Seedling for CDd (P75) Seeds	% Normal Seedling for CDd (P50) Seeds	% Normal Seedling for CDd (P25) Seeds
DW	99.00 ± 1.85 ^NS^	73.50 ± 9.78 ^NS^	50.00 ± 10.03 ^b^	21.00 ± 6.32 ^b^
CaCl_2_	100.00 ± 0.00 ^NS^	70.00 ± 8.28 ^NS^	58.00 ± 11.51 ^NS^	32.00 ± 11.31 ^NS^
CaCl_2_ CW	100.00 ± 0.00 ^NS^	74.00 ± 10.03 ^NS^	67.00 ± 12.42 ^a^	39.00 ± 11.06 ^a^
CaMg	98.50 ± 4.24 ^NS^	76.50 ± 10.57 ^NS^	76.50 ± 10.57 ^a^	40.00 ± 9.56 ^a^
CaMg CW	100.00 ± 0.00 ^NS^	72.50 ± 4.50 ^NS^	63.00 ± 5.95 ^NS^	23.00 ± 6.32 ^NS^
CaMg CW (6.5)	99.00 ± 1.85 ^NS^	76.50 ± 5.83 ^NS^	68.00 ± 10.90 ^a^	29.00 ± 5.13 ^NS^
MgCl_2_	99.00 ± 1.85 ^NS^	83.00 ± 9.50 ^NS^	68.00 ± 10.90 ^NS^	28.00 ± 11.11 ^NS^
MgCl_2_ CW	99.50 ± 1.41 ^NS^	71.00 ± 11.06 ^NS^	57.00 ± 12.96 ^NS^	39.00 ± 10.20 ^a^
NaCl	98.00 ± 2.20 ^NS^	76.25 ± 5.06 ^NS^	50.00 ± 9.07 ^NS^	24.50 ± 6.57 ^NS^
NaCl CW	100.00 ± 0.00 ^NS^	78.50 ± 6.02 ^NS^	51.50 ± 11.99 ^NS^	40.00 ± 14.18 ^a^
NaCl CW (6.5)	99.50 ± 1.41 ^NS^	74.00 ± 6.76 ^NS^	68.00 ± 10.90 ^NS^	48.00 ± 15.57 ^a^

Values represent mean ± SD (4 × *n* = 25) % normal seedling production of the control (fresh seeds soaked in deionised water [DW] and all the inorganic salt solutions), and CDd (P75, P50 and P25) lettuce seeds exposed to DW and inorganic salt solutions. Values labelled with different letters are significantly different (*p* < 0.05, ANOVA) when compared across hydration treatments within each controlled deterioration level. Cathodic water, CW; cathodic water adjusted to pH 6.5, CW (6.5); NS: not significantly different from value obtained with DW and therefore not considered in statistical comparisons.

**Table 4:** Effect of the application of inorganic salt solutions on seedling vigour index of controlled deteriorated (CDd) cabbage and lettuce seeds.

Controlled Deterioration Level	Treatments	Seedling Vigour Index	
		Cabbage	Lettuce
P50	DW	1621.40 ± 911.97 ^b^	1056.90 ± 323.25 ^b^
	CaCl_2_	1564.90 ± 522.56 ^NS^	2705.20 ± 629.67 ^a^
	CaCl_2_ CW	1352.90 ± 797.91 ^NS^	2819.10 ± 580.95 ^a^
	CaMg	2855.00 ± 688.79 ^NS^	2823.50 ± 585.35 ^a^
	CaMg CW	2284.20 ± 781.18 ^NS^	1772.80 ± 362.52 ^NS^
	CaMg CW (6.5)	3395.10 ± 1073.31 ^a^	3493.20 ± 1077.06 ^a^
	MgCl_2_	3042.80 ± 1570.89 ^NS^	2525.20 ± 607.15 ^NS^
	MgCl_2_ CW	2938.70 ± 776.86 ^NS^	2755.80 ± 1094.63 ^a^
	NaCl	2644.10 ± 1514.80 ^NS^	2000.80 ± 510.49 ^NS^
	NaCl CW	2019.30 ± 1042.40 ^NS^	2717.40 ± 906.07 ^a^
	NaCl CW (6.5)	3520.30 ± 910.90 ^a^	2981.20 ± 795.30 ^a^
P25	DW	535.10 ± 222.14 ^b^	403.30 ± 199.74 ^b^
	CaCl_2_	903.00 ± 597.52 ^NS^	1090.93 ± 404.74 ^NS^
	CaCl_2_ CW	1198.40 ± 534.58 ^NS^	1341.80 ± 362.03 ^a^
	CaMg	1679.00 ± 796.61 ^a^	1144.80 ± 405.07 ^NS^
	CaMg CW	531.50 ± 294.91 ^NS^	490.40 ± 296.44 ^NS^
	CaMg CW (6.5)	1084.00 ± 321.11 ^NS^	794.40 ± 395.29 ^NS^
	MgCl_2_	785.50 ± 627.33 ^NS^	747.80 ± 511.32 ^NS^
	MgCl_2_ CW	1084.00 ± 648.73 ^NS^	1205.80 ± 282.89 ^NS^
	NaCl	1438.70 ± 426.89 ^NS^	404.20 ± 147.05 ^NS^
	NaCl CW	1633.10 ± 801.91 ^a^	1493.80 ± 668.57 ^a^
	NaCl CW (6.5)	2205.18 ± 1003.93 ^a^	1562.30 ± 1038.11 ^a^

Values represent mean ± SD (4 × *n* = 25) seedling vigour index (SVI) of CDd (P50 and P25) cabbage and lettuce seeds exposed to DW and inorganic salt solutions. Values labelled with different letters are significantly different (*p* < 0.05, ANOVA) when compared across hydration treatments within each CD level. Cathodic water, CW; cathodic water adjusted to pH 6.5, CW (6.5); controlled deterioration, CD; NS: not significantly different from value obtained with DW and therefore not considered in statistical comparisons.

**Table 5:** Seed pre-hydration treatment solutions used in this study to hydrate fresh and deteriorated seeds of cabbage and lettuce.

S/*n*	Pre-Hydration Treatment Solution	Concentration of Constituent(s)	pH
1	Control	Deionised water	5.6
2	CaCl_2_ (non-electrolysed)	1 mM CaCl_2_	6.0
3	CaCl_2_ generated CW	1 mM CaCl_2_	10.8
4	CaMg (non-electrolysed)	1 µm CaCl_2_; 1 mM MgCl_2_	5.9
5	CaMg generated CW	1 µm CaCl_2_; 1 mM MgCl_2_	11.2
6	CaMg generated CW adjusted to pH 6.5	1 µm CaCl_2_; 1 mM MgCl_2_	6.5
7	MgCl_2_ solution (non-electrolysed)	1 mM MgCl_2_	5.9
8	MgCl_2_ generated CW	1 mM MgCl_2_	10.6
9	NaCl solution (non-electrolysed)	1 mM NaCl	5.6
10	NaCl generated CW	1 mM NaCl	11.2
11	NaCl generated CW adjusted to pH 6.5	1 mM NaCl	6.5

## Supplementary Materials

The following are available online at https://www.mdpi.com/2223-7747/9/9/1164/s1, Figure S1: Water uptake in cabbage and lettuce seeds, Figure S2: Normal seedling production (%) after controlled deterioration of cabbage (A) and lettuce (B) seeds 14 days after germination, Table S1: Effect of the application of inorganic salt solutions on seedling vigour index of fresh and controlled deteriorated (P75) cabbage and lettuce seeds, Table S2: Effect of the application of inorganic salt solutions on mortality (%) in fresh and controlled deteriorated cabbage and lettuce seeds.
