# Supplementary material for: Effects of Inorganic Salt Solutions on Vigour, Viability, Oxidative Metabolism and Germination Enzymes in Aged Cabbage and Lettuce Seeds
Source: Plants (Basel). 2020 Sep 9;9(9):1164. doi: 10.3390/plants9091164 (PMC7569860; doi:10.3390/plants9091164)
Supplement: Supplementary file 1 [file plants-09-01164-s001.pdf]

Supplementary data

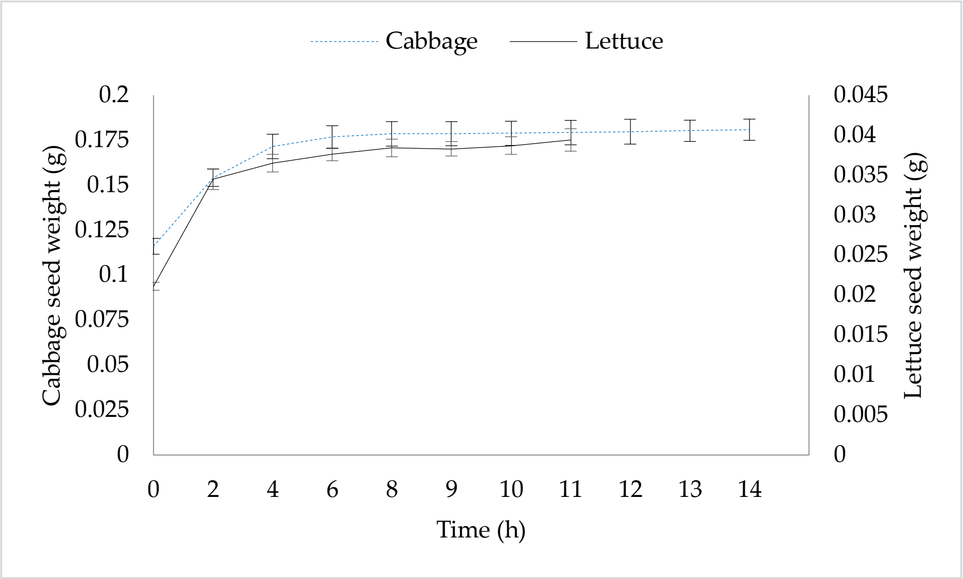

Figure S1. Water uptake in cabbage and lettuce seeds. Data points represent mean±SD (3 experiments of n = 25 seeds for each trial).

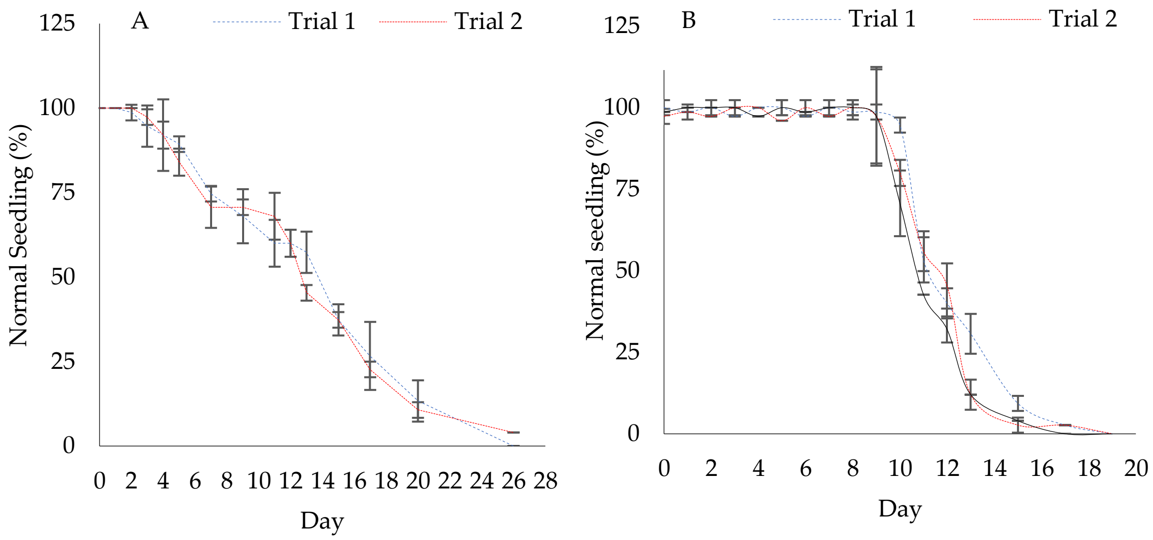

Figure S2. Normal seedling production (%) after controlled deterioration of cabbage (A) and lettuce (B) seeds 14 days after germination. Data points represent mean±SD (4 experiments of n = 25 seeds for each trial).

Table S1. Effect of the application of inorganic salt solutions on seedling vigour index of fresh and controlled deteriorated (P75) cabbage and lettuce seeds.

**Controlled deterioration Level**	Treatments	Seedling Vigour Index	
		Cabbage	Lettuce
**Fresh**	DW	6721.20±946.967^{NS}	3446.30±530.44^{NS}
	$CaCl_2$	6820.20± 912.16^{NS}	3655.00±457.42^{NS}
	$CaCl_2$ CW	6542.10±1369.35^{NS}	3525.00±244.66^{NS}
	CaMg	6284.90±1346.14^{NS}	3749.80±474.16^{NS}
	CaMg CW	5917.50±1075.33^{NS}	3462.50±289.32^{NS}
	CaMg CW (6.5)	7173.20±1231.64^{NS}	3440.50±333.14^{NS}
	$MgCl_2$	7004.70±888.17^{NS}	3647.00±414.42^{NS}
	$MgCl_2$ CW	6752.80±765.97^{NS}	3404.50±340.25^{NS}
	NaCl	4692.50±1904.47^{NS}	3431.95±231.65^{NS}
	NaCl CW	6906.30±841.71^{NS}	3400.00±153.44^{NS}
	NaCl CW (6.5)	6060.50±1158.96^{NS}	3463.90±482.66^{NS}
**P75**	DW	7554.50±2623.67^{NS}	4191.90±842.42^{NS}
	$CaCl_2$	5559.45±1833.26^{NS}	3560.70±7181.02^{NS}
	$CaCl_2$ CW	7126.30±2643.67^{NS}	4387.50±1315.91^{NS}
	CaMg	4357.00±1044.53^{NS}	4047.90±1256.79^{NS}
	CaMg CW	6512.00±1503.23^{NS}	4157.10±328.56^{NS}
	CaMg CW (6.5)	6627.20±2031.09^{NS}	4512.70±673.33^{NS}
	$MgCl_2$	8012.50±2757.45^{NS}	4918.10±831.24^{NS}
	$MgCl_2$ CW	6612.50±1427.34^{NS}	3552.20±880.11^{NS}
	NaCl	4672.20±2007.78^{NS}	4491.45±782.45^{NS}
	NaCl CW	6359.80±1727.57^{NS}	4592.30±1117.40^{NS}
	NaCl CW (6.5)	6435.00±2780.75^{NS}	4390.60±882.63^{NS}

Values represent mean±SD (4 x n = 25) of inorganic salt solutions that enhanced seedling vigour index (SVI) significantly relative to DW-treated seeds in fresh and P75. Values labelled with different letters are significantly different ($P < 0.05$, ANOVA) when compared across hydration treatments within each CD level. Cathodic water, CW; cathodic water adjusted to pH 6.5, CW (6.5); controlled deterioration, CD; NS: not significantly different from value obtained with DW and therefore not considered in statistical comparisons.

Table S2. Effect of the application of inorganic salt solutions on mortality (%) in fresh and controlled deteriorated cabbage and lettuce seeds.

Hydration treatments	Cabbage				Lettuce			
	**% mortality for fresh seeds**	**% mortality for CDd (P75) seeds**	**% mortality for CDd (P50) seeds**	**% mortality for CDd (P25) seeds**	**% mortality for fresh seeds**	% mortality for **CDd** (P75) seeds	% mortality for **CDd** (P50) seeds	% mortality for **CDd** (P25) seeds
DW	11.00±9.26[NS]	17.00±8.21[NS]	38.50±5.63[b]	60.00±3.02[b]	1.00±1.85[NS]	20.00±7.09[NS]	38.50±11.70[a]	70.00±5.24[a]
$CaCl_2$	6.00±6.76[NS]	23.50±6.57[NS]	65.00±7.33[a]	70.50±11.10[a]	0.00±0.00[NS]	23.00±5.95[NS]	33.00±8.75[NS]	55.00±11.66[NS]
$CaCl_2$ CW	5.50±5.21[NS]	20.00±7.09[NS]	71.00 ±12.78[a]	73.50±5.21[a]	0.00±0.00[NS]	23.00±7.63[NS]	29.00±7.93[NS]	54.00±11.51[NS]
CaMg	7.50±7.54[NS]	30.00±4.28[NS]	51.50±6.91[NS]	68.50±4.50[a]	1.50±4.24[NS]	20.50±6.57[NS]	13.00±5.13[b]	51.50±13.60[b]
CaMg CW	8.50±9.43[NS]	22.50±7.98[NS]	33.00±11.46[NS]	72.00±3.70[a]	0.00±0.00[NS]	24.00±4.28[NS]	31.50±10.13[NS]	66.00±10.03[NS]
CaMg CW (6.5)	3.50±5.83[NS]	23.50±7.23[NS]	38.00±7.09[NS]	63.50±2.56[NS]	1.00±1.85[NS]	20.50±3.96[NS]	28.00±11.90[NS]	63.00±9.74[NS]
$MgCl_2$	5.00±7.01[NS]	15.50±10.13[NS]	54.50±8.80[NS]	77.50±6.39[a]	1.00±1.85[NS]	13.00±7.33[NS]	36.50±7.84[NS]	60.00±6.41[NS]
$MgCl_2$ CW	7.50±9.90[NS]	17.50±6.74[NS]	46.50±6.02[NS]	64.50±9.67[NS]	0.50±1.41[NS]	27.50±11.80[NS]	36.50±5.42[NS]	50.00±11.11[b]
NaCl	6.50±5.47[NS]	25.00±12.42[NS]	28.50±11.40[NS]	70.75±3.01[a]	1.50±1.85[NS]	21.00±5.55[NS]	32.00±12.28[NS]	56.50±13.43[NS]
NaCl CW	4.50±4.99[NS]	15.50±6.21[NS]	57.00±13.31[a]	65.50±4.75[NS]	0.00±0.00[NS]	21.00±5.95[NS]	40.00±10.03[NS]	49.50±10.03[b]
NaCl CW (6.5)	6.00±8.00[NS]	22.50±7.69[NS]	46.50±4.24[NS]	59.00±7.33[NS]	0.50±1.41[NS]	21.00±5.55[NS]	36.00±5.66[NS]	47.00±8.75[b]

Values represent mean±SD (4 x n = 25) % mortality of the control (fresh seeds soaked in deionised water [DW] and all the inorganic salt solutions), and CDd (P75, P50 and P25) cabbage and lettuce seeds exposed to the inorganic salt solutions. Values labelled with different letters are significantly different ($P < 0.05$, ANOVA) when compared across hydration treatments within each controlled deterioration level. Cathodic water, CW; cathodic water adjusted to pH 6.5, CW (6.5); controlled deteriorated, CDd; not significant, NS.

© 2020 by the authors. Submitted for possible open access publication under the terms and conditions of the Creative Commons Attribution (CC BY) license (http://creativecommons.org/licenses/by/4.0/).
